# Adenovirus 7 Induces Interlukin-6 Expression in Human Airway Epithelial Cells *via* p38/NF-κB Signaling Pathway

**DOI:** 10.3389/fimmu.2020.551413

**Published:** 2020-09-23

**Authors:** Lifeng Qi, Yajuan Wang, Heping Wang, Jikui Deng

**Affiliations:** ^1^Department of Neonatology, Beijing Children's Hospital, Capital Medical University, National Center for Children's Health, Beijing, China; ^2^Department of Infectious Disease, Shenzhen Children's Hospital, Shenzhen, China; ^3^Department of Neonatology, Children's Hospital, Capital Institute of Pediatrics, Beijing, China; ^4^Department of Respiratory Diseases, Shenzhen Children's Hospital, Shenzhen, China

**Keywords:** p38/NF-κB signaling pathway, airway epithelial cell, Interlukin-6, inflammation, Adenovirus 7

## Abstract

Human Adenovirus (AdV) infection is very common and usually has a significant impact on children. AdV-induced inflammation is believed to be one of the main causes of severe symptoms. However, an inflammatory response profile in the airway in AdV-infected children is still lacking, and the mechanism underlying AdV-induced inflammation in the airway is also poorly understood. In the current study, we determined the expression of a panel of inflammation cytokines in the airway samples from AdV 7 infected children and further investigated the molecular mechanism underlying AdV 7-induced cytokine expression. Our results showed that eight out of 13 tested inflammatory cytokines were significantly increased in nasal washes of AdV 7-infected children comparing to healthy control, with IL-6 showing the highest enhancement. AdV 7 infection of bronchial epithelial cell line and primary airway epithelial cells confirmed that AdV 7 increased IL-6 mRNA and protein expression in an infection dose-dependent manner. Promoter analysis revealed that AdV 7 infection transactivated IL-6 promoter and a NF-κB binding site in IL-6 promoter was involved in the transactivation. Further analysis showed that upon AdV 7 infection, NF-κB p65 was phosphorylated and translocated into nucleus and bound onto IL-6 promoter. Signaling pathway analysis revealed that p38/NF-κB pathway was involved in AdV 7 infection induced IL-6 elevation. Taken together, our study shows that AdV 7 infection triggers the expression of a range of inflammatory cytokines including IL-6 in the airway of infected children, and AdV 7 enhances IL-6 expression by transactivating IL-6 promoter *via* p38/NF-κB signaling pathway. Findings of our current study have provided more information toward a better understanding of AdV-induced airway inflammation, which might also benefit the development of intervention strategies.

## Introduction

Human Adenovirus (AdV) infection is very common and has a significant impact on children (1). The clinical manifestations of AdV infection are diverse and usually virus strain specific. While the symptoms are usually respiratory, they can sometimes also be ocular and gastrointestinal tract symptoms (2, 3). AdV infection usually causes mild symptoms, but it can also lead to severe respiratory and gastrointestinal damage and even death (3–6). There are a few known risk factors that are associated with severe adenovirus-related disease, and children of age <7 years old is one of them (7). Therefore, this highly transmissible AdV has significant impact on the health of young children and to understand the pathogenesis of AdV infection is of importance for the prevention and treatment of the infection.

Up to now, there are more than 60 AdV serotypes that have been identified, and they can be divided into seven subgroups (group A–G) based on DNA sequence similarity, biophysical and biochemical properties (8). Usually, each AdV subgroup can have distinctive clinical symptoms. For example, subgroup B1, C, and E AdVs tend to cause respiratory disease, subgroup D usually leads to keratoconjunctivitis, whereas subgroup F is often associated with gastroenteritis. The serotypes that are usually associated with infection in children are subgroup B (AdV 3 and 7), subgroups C (AdV 1, 2, 5 and 6), and subgroup E (AdV 4) (9, 10). Among them, AdV 3, 4, and 7 are more likely to result in severe infections (10, 11). AdV 7, in particular, has been proven to be a cause of pneumonia in children (6, 12).

Although detailed mechanism underlying AdV infection caused clinical symptoms still remain to be fully elucidated, the infection induced inflammation is believed to be one of the main causes (13). AdV is a pro-inflammatory virus that can trigger high level of host innate responses, including secretion of inflammatory cytokines and chemokines. While appropriate magnitude of inflammatory response is needed for the host to clear infection, excess inflammation, on the other hand, will lead to serious damage to the host (14). Studies are still underway to characterize AdV-caused innate inflammatory responses, to understand the underlying molecular mechanisms and to explore ways to balance the response toward viral clearance with minimal host tissue damage. A few pro-inflammatory cytokines, including IL-8 and TNF-α, has been shown to be elevated upon AdV infection in both *in vitro* and *in vivo* studies (2, 15–17). However, a more detailed inflammatory response in children with AdV infection is still lacking, and moreover, the mechanism underlying AdV-infection induced inflammation in the airway is still poorly understood.

In the current study, we focused on AdV 7, the serotype causing pneumonia in children, and investigated the inflammatory cytokine profile of children with AdV 7 infection and the underling molecular mechanism using both airway epithelial cell line and primary airway epithelial cells. The findings of our current study have not only provided a more comprehensive inflammation cytokine profile for airway AdV infection, but also elucidated a detailed signaling pathway involved in the viral infection-induced inflammation. The information will be beneficial for the understanding of AdV pathogenesis as well as the development of preventive and treatment strategies.

## Materials and Methods

### Ethical Statement

This study was carried out in accordance with the recommendations of the Ethics committee of Shenzhen Children's Hospital. The protocol was approved by the Ethics Committee of Shenzhen Children's Hospital. Informed consent was obtained from the guidance of the participants in accordance with the Declaration of Helsinki.

### Patients and Controls Enrollment

All participants in the current study were enrolled between March 2018 and November 2019. A total number of 26 (13 male; 13 female) AdV 7 positive patients were enrolled based on the following criteria: (1) AdV 7 positive by PCR; (2) Age no older than seven; (3) Typical respiratory infection symptoms including fever, cough, and rapid breathing. In addition, 10 (five male; five female) controls were also recruited based on the following criteria: (1) AdV 7 negative by PCR; (2) Age no older than seven; (3) Absence of typical respiratory infection symptoms. After informed consent was obtained, a nasal wash sample was collected from each participant and kept frozen at −80°C until analysis.

### Cells and Plasmids

Normal bronchial epithelial cell line BEAS-2B, human cervical epithelial cell line HeLa and primary human airway epithelial cells (hAEC) were used in the current study. All cells were purchased from ATCC and cultured according to the ATCC recommendations.

Full length IL-6 promoter-controlled luciferase reporter gene plasmid, (-1,000/+11)IL-6-Luc, was constructed by inserting the−1,000 bp to +11 pb sequence upstream of human IL-6 coding sequence into pGL3-Basic vector (Promega). Truncated and site-directed mutated IL-6 promoter-controlled luciferase reporter gene plasmids were made based on the full length plasmid (-1,000/+11)IL-6-Luc. All plasmids were verified by sequencing.

### Inflammatory Cytokine Profiling

Inflammatory cytokines IL-1β, IFN-α2, IFN-γ, TNF-α, MCP-1, IL-6, IL-8, IL-10, IL-12p70, IL-17A, IL-18, IL-23, and IL-33 were measured in the nasal wash samples collected from patients and controls by BioLegend's LEGENDplex™ bead-based immunoassay, according to the manufacturer's instructions (BioLegend). In brief, samples and standards were thawed at room temperature and then incubated with mixed capture beads in a filter plate on a shaking platform for 2 h at room temperature. Following incubation, the plate was washed and sequentially incubated with detection antibodies and SA-PE for 1 h and 30 min, respectively. After final washes, the beads were resuspended in wash buffer and transferred into FACS tubes. Samples were then read on a BD LSRFortessa flow cytometer (BD Biosciences). Data was analyzed using the LEGENDplex Data Analysis Software according to the manufacturer's instructions (BioLegend).

### AdV Propagation and Titration

AdV 7 (Gomen strain) was purchased from ATCC and propagated and titrated according to ATCC recommendations. In brief, AdV 7 was inoculated into 70–80% confluent HeLa cell monolayer and culture for 3–5 days. The virus was then harvested and purified using a standard cesium chloride gradient centrifugation procedure, as described previously (18). Purified virus was then quantified on HeLa cells using the Light Diagnostics Adenovirus DFA Kit (Merck Millipore), according to the manufacturer's instructions.

### AdV Infection

BEAS-2B cells and primary human airway epithelial cells were used for AdV infection in the current study. For viral dose assay, cells were infected with ascendant doses of AdV, ranging from 0 to 10 MOI. For other studies, cells were infected with 1 MOI of AdV 7. Cells were first inoculated with AdV 7 for 1 h at 37°C, and then inoculum was removed, and cells were washed and cultured in complete medium for another 24 h. Following culture, cells and cell culture supernatants were harvested for analysis. For cells requiring both viral infection and DNA transfection, viral infection was performed at 4–6 h post transfection.

### DNA and siRNA Transfection and Inhibitor Treatment

All DNA transfections were performed using Lipofectamine 2000, according to the manufacturer's instructions (Thermo Scientific Invitrogen). All siRNA transfections were performed using X-tremeGENE siRNA Transfection Reagent (Roche), according to the manufacturer's instructions. For signaling inhibitor treatment, cells were treated with PD98059 (MEK inhibitor), SB203580 (p38 inhibitor), SP600125 (JNK inhibitor) and BAY11-7082 (NF-κB inhibitor), 1 h post AdV 7 infection, and remained in the culture until analysis. All signaling pathway inhibitors were purchased from InvivoGen and used according to the manufacturer's instructions.

### Luciferase Reporter Gene Assay

The luciferase reporter gene assay was used to evaluate AdV 7-induced IL-6 promoter activation. The assay was performed as previously described with modifications (19, 20). In brief, cells were first transfected with full length IL-6 promoter-controlled luciferase reporter gene plasmid, (-1,000/+11)IL-6-Luc, or its truncations or mutations, together with the Renilla-expression control plasmid pRL-TK. Four to 6 h post transfection, cells were either mock infected, or infected with AdV 7. Twenty-four hours later, cells were lysed and luciferase activity was quantified using the Dual Luciferase Assay Kit (Promega), according to the manufacturer's instructions. For cells requiring siRNA treatment, siRNA transfection was done 24 h before plasmid transfection. For cells requiring signaling pathway inhibition, specific signaling pathway inhibitors were introduced 1 h after AdV 7 infection and maintained in the culture until luciferase measurement.

### RT-PCR

IL-6 mRNA level quantification was done by semi-quantitative RT-PCR, as previously described with modifications (21). In brief, total RNA was extracted from cells using RNeasy Mini Kit (Qiagen) according to the manufacturer's instructions. Then, mRNA was reverse transcribed into cDNA using PrimeScript 1st strand cDNA Synthesis Kit (TaKaRa) according to the manufacturer's instructions. IL-6 mRNA level was then measured by SYBR Green RT-PCR using SsoAdvanced™ Universal SYBR Green Supermix (Bio-Rad) on a Bio-Rad CFX connect platform. Relative IL-6 mRNA level was calculated using the 2^−ΔΔ*Ct*^ method, with GAPDH as an internal control. The following primer pairs were used: IL-6 forward primer: 5′-ATGAACTCCTTCTCCACAAGC-3′, and IL-6 reverse primer: 5′-GTTTTCTGCCAGTGCCTCTTTG-3′; GAPDH forward primer: 5′- GCCAAGGTCATCCATGACAACTTTGG-3′, and GAPDH reverse primer: 5′- GCCTGCTTCACCACCTTCTTGATGTC-3′.

### ELISA

IL-6 concentration in cell culture supernatants was quantified by ELISA, using Human IL-6 DuoSet ELISA Kit (R&D Systems), according to the manufacturer's instructions. In brief, Nunc MaxiSorp™ flat-bottom ELISA plate (Thermo Fisher Scientific) was first coated with capture antibody overnight at room temperature, and then washed and blocked for 1 h at room temperature. Following washes, plate was sequentially incubated with samples or standards, detection antibody and Streptavidin-HRP for 2 h, 2 h, and 20 min, respectively. After extensive washes, TMB substrate solution was added and incubated for 20 min at room temperature and followed by the addition of Stop solution. Plate was then read on an ELISA plate reader (Tecan) using a test wavelength of 450 nm and a reference wavelength of 570 nm. IL-6 concentration was then calculated from the standard curve generated using the four-parameter logistic regression.

### Western Blot

Western blot was performed as previously described with modifications (19). In brief, cells were lysed with cell lysis buffer in the presence of protease inhibitor cocktail and phosphatase cocktail (both from Abcam) and mixed with SDS-PAGE loading buffer. For Western blot analysis of subcellular fractions, cell cytoplasmic and nuclear proteins were first fractionized by Cell Fractionation Kit (Abcam), according to the manufacturer's instructions, and then each fraction was mixed with SDS-PAGE loading buffer. Prepared samples were then separated by 12% SDS-PAGE gel and transferred onto a PVDF membrane. Following blocking with 5% non-fat milk for 1 h at room temperature, the membrane was then sequentially incubated with primary antibodies and secondary antibodies for overnight at 4°C and 1 h at room temperature, respectively. After extensive washes, the membrane was then developed by ECL substrate (Thermo Fisher Scientific) and imaged by a CCD camera (Bio-Rad). The following primary antibodies were used: mouse anti-human p65 (Santa Cruz), rabbit anti-human p-p65 (Cell Signaling Technology), mouse anti-human GAPDH (Santa Cruz), rabbit anti-human PCNA (Proteintech), rabbit anti-human p38 (Cell Signaling Technology), rabbit anti-human p-p38 (Cell Signaling Technology). The following secondary antibodies were used: goat anti-mouse IgG(H+L)-HRP and goat anti-rabbit IgG(H+L)-HRP (both from Boster Biotechnology).

### Chromatin Immunoprecipitation (ChIP)

ChIP assay was performed using a Pierce Magnetic ChIP Kit (Thermo Fisher Scientific) according to the manufacturer's instructions. The following ChIP grade antibodies were used: mouse anti-human p65 and normal mouse IgG (negative control). The following primer pairs were used to amplify p65 promoter specific fragment from ChIP purified DNA: forward primer: 5′-GACGACCCCAATTCAAATCG-3′ and reverse primer: 5′-TCAGGCTCGGGGAATTTCC-3′. The antibodies and primers were purchased from Merck Millipore and used according to the manufacturer's instructions.

### Statistical Analysis

All the statistical analyses in the current study were conducted with GraphPad Prism 8.4.1. For comparisons between two groups, a Mann-Whitney test was used, and for comparisons among three or more groups, Kruskal-Wallis test with Dunn's multiple comparisons test was used. A *p* < 0.05 was considered statistically significant.

## Results

### AdV 7 Infection Triggers the Production of Inflammatory Cytokines in the Airway in Children

**Figure 1:**
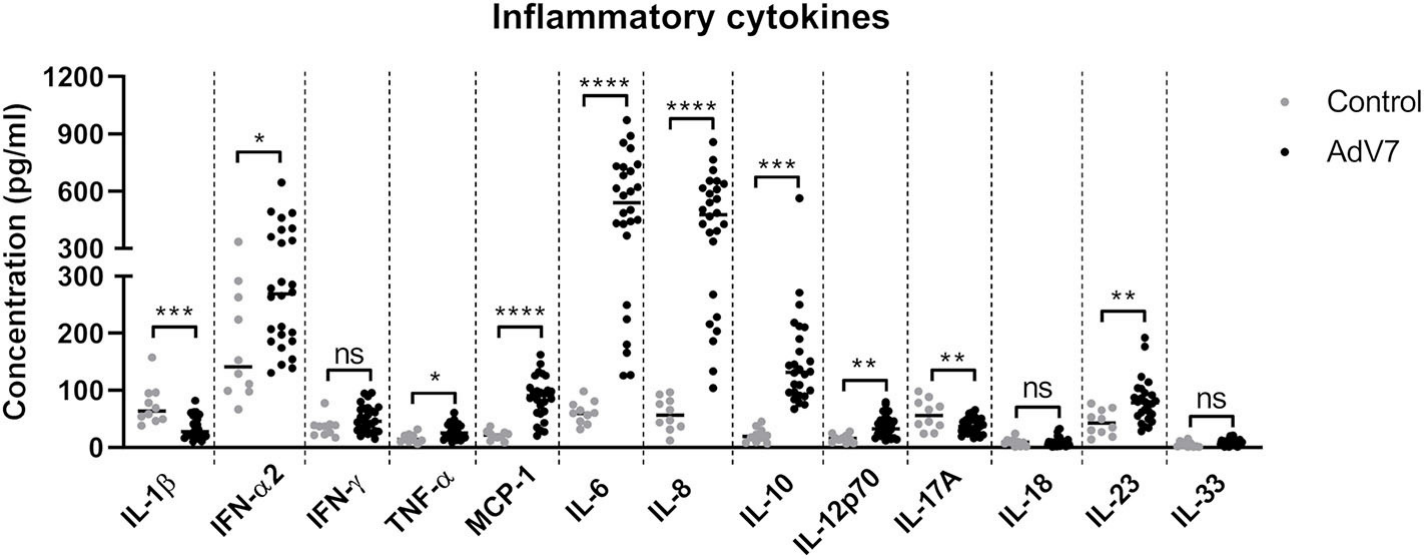
AdV 7 infection triggers the production of inflammatory cytokines in the airway in children. Nasal samples from AdV 7 infected and healthy children were harvested and inflammatory cytokines IL-1β, IFN-α2, IFN-γ, TNF-α, MCP-1, IL-6, IL-8, IL-10, IL-12p70, IL-17A, IL-18, IL-23, and IL-33 were measured by BioLegend's LEGENDplex bead-based immunoassay. ns, statistically not significant; *p < 0.05; **p < 0.01; ***p < 0.001; ****p < 0.0001.

To better understand how AdV 7 infection impacts the inflammatory response in the respiratory system, we collected nasal wash samples from AdV 7-positive and -negative children under the age of seven and a panel of 13 inflammatory cytokines were quantified. As shown in Figure 1, AdV 7 as a pro-inflammatory virus significantly elevated the expression of many inflammatory cytokines. Of the 13 cytokines measured, 8 (IFN-α2, TNF-α, MCP-1, IL-6, IL-8, IL-10, IL-12p70, and IL-23) were significantly increased comparing to control group. Of note, IL-6 showed the highest increase upon AdV 7 infection among the eight increased cytokines. To investigate the possible mechanism underlying AdV 7 infection induced inflammation, we in the current study chose IL-6 as a representative inflammatory effector and further explored its expression in relation to AdV 7 infection.

### AdV 7 Induces IL-6 mRNA and Protein Expression in Airway Epithelial Cells

**Figure 2:**
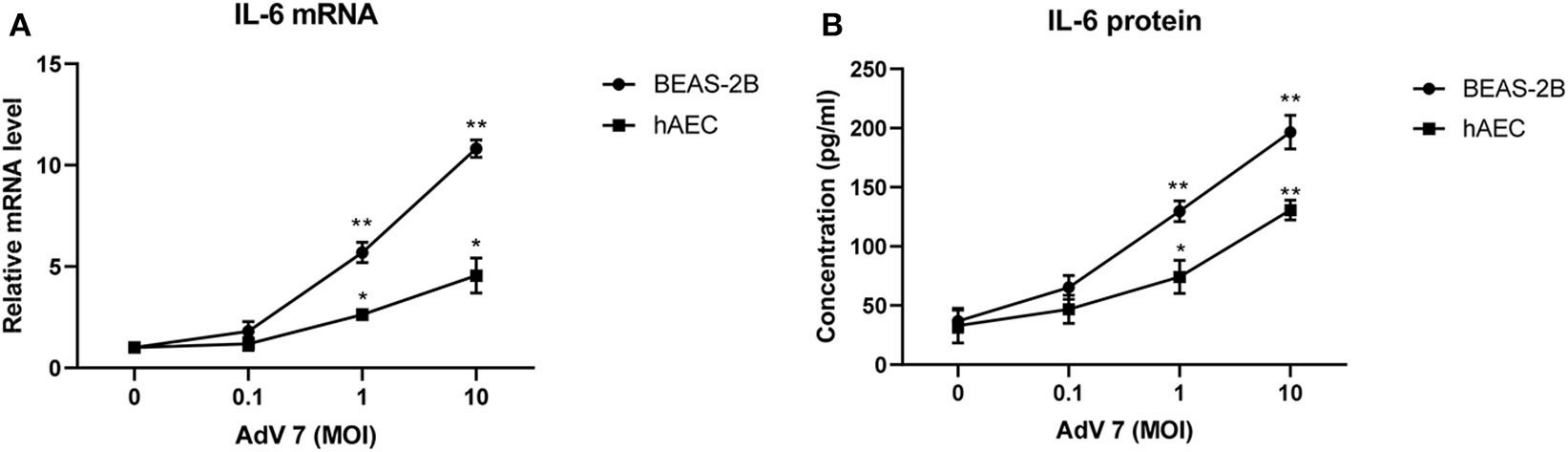
AdV 7 infection induces IL-6 mRNA and protein expression in airway epithelial cells. Human normal bronchial epithelial cell line BEAS-2B and primary human airway epithelial cells (hAEC) were either mock-infected or infected with ascendant doses of AdV 7 for 24 h and then **(A)** IL-6 mRNA and **(B)** protein expression were measured by RT-PCR and ELISA, respectively. Data shown are mean ± *SD* of three independent experiments with each condition performed in duplicate. *p < 0.05; **p < 0.01.

We chose normal bronchial epithelial cell line BEAS-2B and primary human airway epithelial cells (hAEC) as *in vitro* cell models. To confirm that the AdV 7-induced IL-6 elevation observed in patients' samples could be reproduced *in vitro*, BEAS-2B cells and hAEC cells were first infected with ascendant doses of AdV 7 for 24 h, and then IL-6 mRNA and protein levels were quantified. As shown in Figure 2, both BEAS-2B and hAEC cells showed increased IL-6 expression on both mRNA (Figure 2A) and protein levels (Figure 2B) upon AdV 7 infection, and such increase was more profound in BEAS-2B cells than in hAEC cells. In addition, the IL-6 mRNA and protein levels increased in an AdV 7 infection dose dependent manner (Figure 2). After infection of AdV 7 ranging from 0 to 10 MOI for 24 h, cells did not show apparent cytopathic effect or cell death (unpublished data and Supplementary Figure 1). Since AdV 7 at 1 MOI induced significantly higher IL-6 expression comparing to cells without infection, we chose 1 MOI AdV 7 for infection for the downstream experiments.

### A NF-κB Binding Site in the IL-6 Promoter Is Involved in AdV 7-Induced IL-6 Transactivation

**Figure 3:**
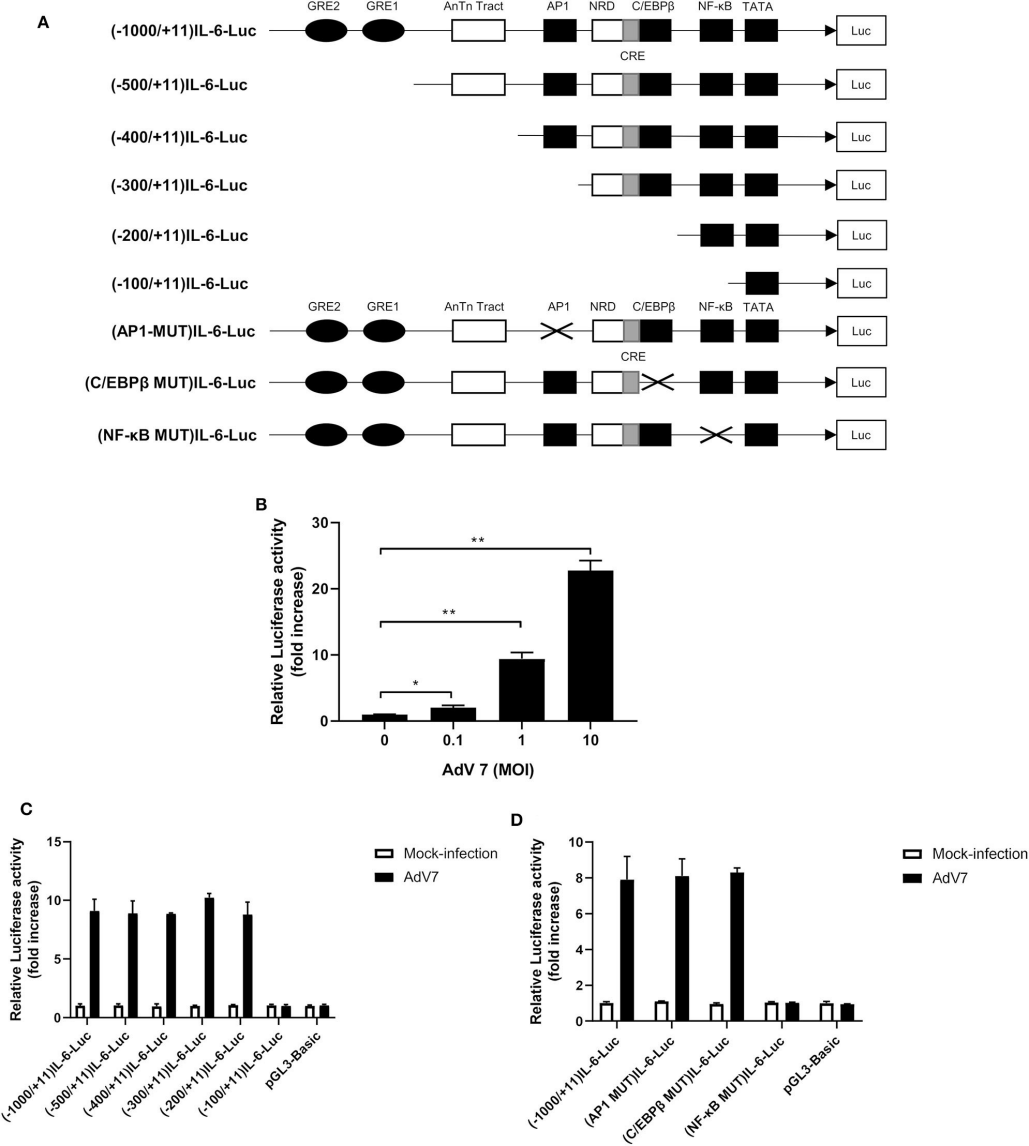
A NF-κB binding site in the IL-6 promoter is involved in AdV 7-induced IL-6 transactivation. **(A)** Schematic illustration of all the IL-6 promoter-controlled luciferase reporter gene plasmids. **(B)** BEAS-2B cells were first transfected with (-1,000/+11)IL-6-Luc, and then either mock-infected or infected with ascendant doses of AdV 7 for 24 h. Afterwards, cells were lysed and luciferase activity was measured. Data shown are mean ± *SD* of three independent experiments with each condition performed in duplicate. *p < 0.05; **p < 0.01. **(C,D)** BEAS-2B cells were first infected with either **(C)** serially truncated IL-6 promoter plasmids or **(D)** site-directed mutated IL-6 promoter plasmids, and then were either mock-infected or infected with 1 MOI of AdV 7. Twenty-four hours later, cells were lysed, and luciferase activity was measure. Data shown are mean ± *SD* of three independent experiments with each condition performed in duplicate.

Since AdV 7 infection enhanced IL-6 mRNA level (Figure 2A), we wondered if AdV 7-induced IL-6 expression was due to enhanced IL-6 promoter transactivation. To test our hypothesis, we constructed a luciferase-coding plasmid under the control of human IL-6 promoter, namely (-1,000/+11)IL-6-Luc, and tested its response to AdV 7 infection (Figure 3A). BEAS-2B cells were first transfected with (-1,000/+11)IL-6-Luc, and then mock-infected, or infected with 0.1, 1, and 10 MOI of AdV 7. Measurement of luciferase activity showed that luciferase expression was significantly increased when cells were infected with AdV 7 (Figure 3B). Furthermore, such luciferase increase was shown in an AdV 7 infection dose dependent manner (Figure 3B), indicating that AdV 7 can transactivate IL-6 promoter.

Transactivation of promoter depends on certain transcriptional factors binding onto specific binding sites in the promoter sequence. In order to identify the transcriptional binding site that is involved in the AdV7-induced IL-6 promoter transactivation, we did a serial 5' flanking truncations on the IL-6 promoter and tested their response to AdV 7 infection (Figure 3A). As shown in Figure 3C, the deletion of −1,000 to −200 bp did not apparently affect AdV 7-induced luciferase expression, while the deletion of −200 to −100 bp almost completely diminished the luciferase signal, indicating that −200 to −100 bp region in the IL-6 promoter is essential for the AdV 7 induced IL-6 promoter transactivation. Previous bioinformatics analysis showed that there are a few transcriptional factor binding sites in the region, including AP1, C/EBPβ, and NF-κB (22).

To confirm which transcriptional factor binding site is involved in AdV 7-induced promoter transactivation, we introduced site-specific mutations into (-1,000/+11)IL-6-Luc to remove the individual binding site (Figure 3A). Luciferase measurement showed that only the mutation of NF-κB binding site, but not AP1 and C/EBPβ, resulted in the loss of luciferase signal, indicating that NF-κB binding site in IL-6 promoter is involved in AdV 7-induced promoter transactivation (Figure 3D).

### AdV 7 Infection Induces NF-κB Binding Onto IL-6 Promoter

**Figure 4:**
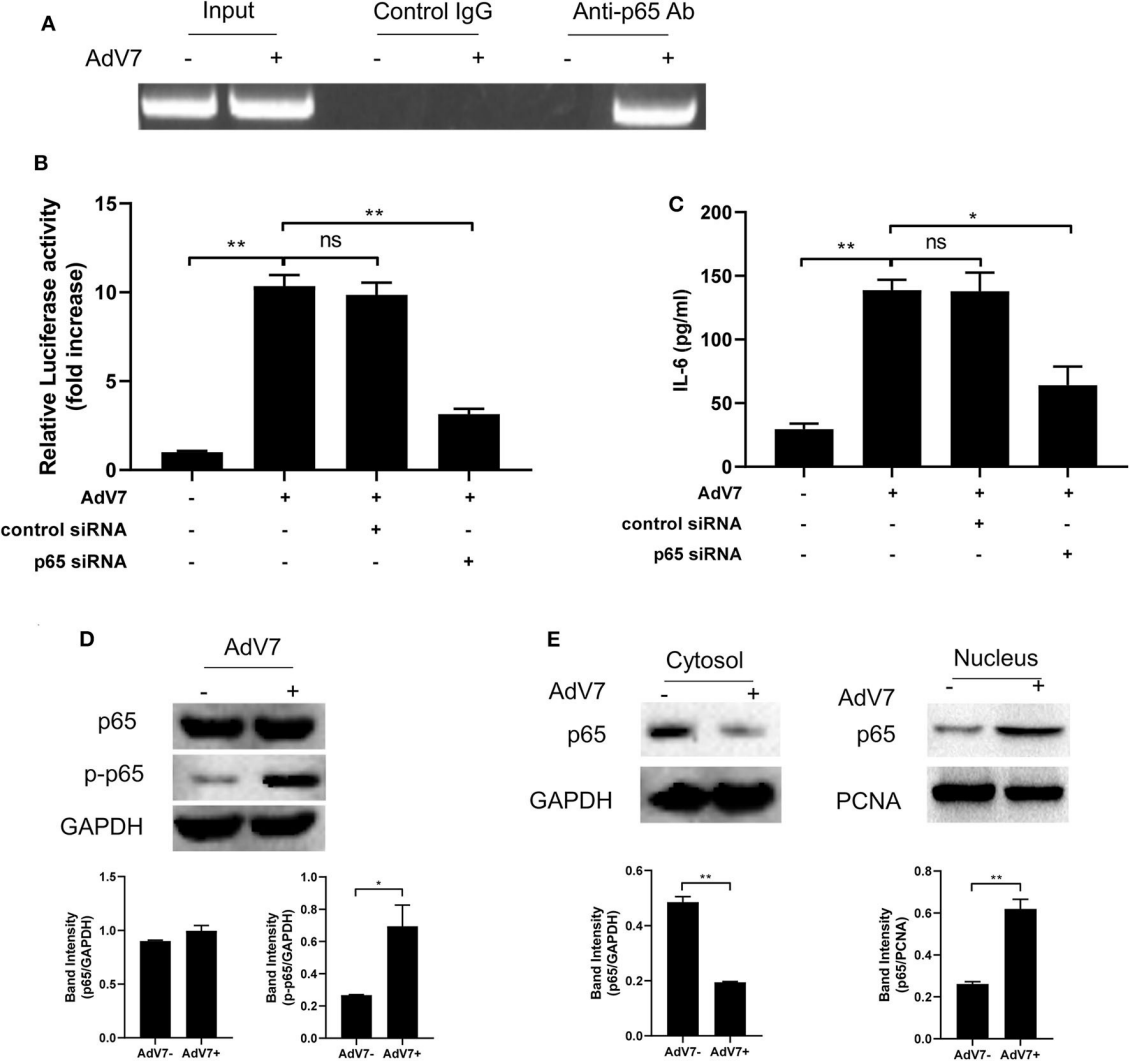
AdV 7 infection induces NF-κB binding onto IL-6 promoter. **(A)** BEAS-2B cells were either mock-infected or infected with 1 MOI AdV 7 for 24 h, and then ChIP assay was performed with either control IgG or anti-p65 antibody. The amplification of IL-6 promoter sequence from ChIP-pulldown was performed with PCR. One representative result is shown. **(B)** BEAS-2B cells were sequentially transfected with control siRNA or p65 siRNA and (-1,000/+11)IL-6-Luc, and then were mock-infected or infected with 1 MOI AdV 7. Twenty-four hours later, cells were lysed, and luciferase activity was measured. Data shown are mean ± *SD* of three independent experiments with each condition performed in duplicate. Ns, not statistically significant; **p < 0.01. **(C)** BEAS-2B cells were first transfected with either control siRNA or p65 siRNA, and then were mock-infected or infected with 1 MOI AdV 7. Twenty-four hours later, IL-6 concentration in the cell culture supernatant was quantified by ELISA. Data shown are mean ± *SD* of three independent experiments with each condition performed in duplicate. Ns, not statistically significant; *p < 0.05; **p < 0.01. **(D,E)** BEAS-2B cells were either mock-infected or infected with 1 MOI AdV 7, and then **(D)** cells were lysed, or **(E)** cell cytoplasmic and nuclear fractions were isolated, and then **(D,E)** the expression of p65 and p-p65 was determined by Western blot. One out of three results is sown.

ChIP assay was performed to further confirm that NF-κB can bind onto IL-6 promoter. BEAS-2B cells were first mock-infected or infected with AdV 7, and then chromatin was segmented and pulled down by control IgG or anti-NF-κB p65 IgG, and IL-6 promoter specific fragment was amplified from the pulldown by PCR. As shown in Figure 4A, a positive band was only detected in the AdV 7 infected cells pulled down by anti-p65 antibody, but not in either mock-infected samples or control IgG pulled down samples, indicating that upon AdV 7 infection, NF-κB can bind onto IL-6 promoter.

To further investigate the importance of NF-κB in AdV 7 induced IL-6 expression, NF-κB p65 was first knocked down with siRNA and then AdV 7 infection induced IL-6 promoter transactivation and IL-6 protein level were determined. Our data showed that in cells with p65 siRNA treatment, IL-6 promoter activation was significantly reduced (Figure 4B). Measurement of IL-6 concentration in the cell culture supernatant also showed a similar reduction in cells with p65 knockdown (Figure 4C). These data further confirmed that NF-κB is involved in the AdV 7 regulation of IL-6 expression.

As a transcriptional factor complex, NF-κB is a heterodimer of p65 and p50. Under inactivated status, NF-κB locates in the cytoplasm and is in association with an inhibitory protein IκB. Upon stimulation, IκB is degraded and NF-κB is phosphorylated and activated. The activated NF-κB will then translocate into nucleus and bind to specific binding site(s) of certain promoters and regulate the transcription of target genes. We next investigated whether AdV 7 infection had impact on the expression, phosphorylation and subcellular location of NF-κB. As shown in Figure 4D, AdV 7 infection did not show apparent change on NF-κB p65 expression, but considerably increased its phosphorylation level. Further isolation of cytoplasmic and nuclear fractions revealed that NF-κB p65 mainly located in the cytoplasm in mock-treated cells, but upon AdV 7 infection, an apparent shift of p65 from the cytoplasm into nucleus was observed (Figure 4E). These data here indicate that AdV 7 infection does not affect the expression of NF-κB, but instead increases its phosphorylation and nuclear translocation.

### AdV 7 Infection Induces IL-6 Expression *via* p38/NF-κB Signaling Pathway

**Figure 5:**
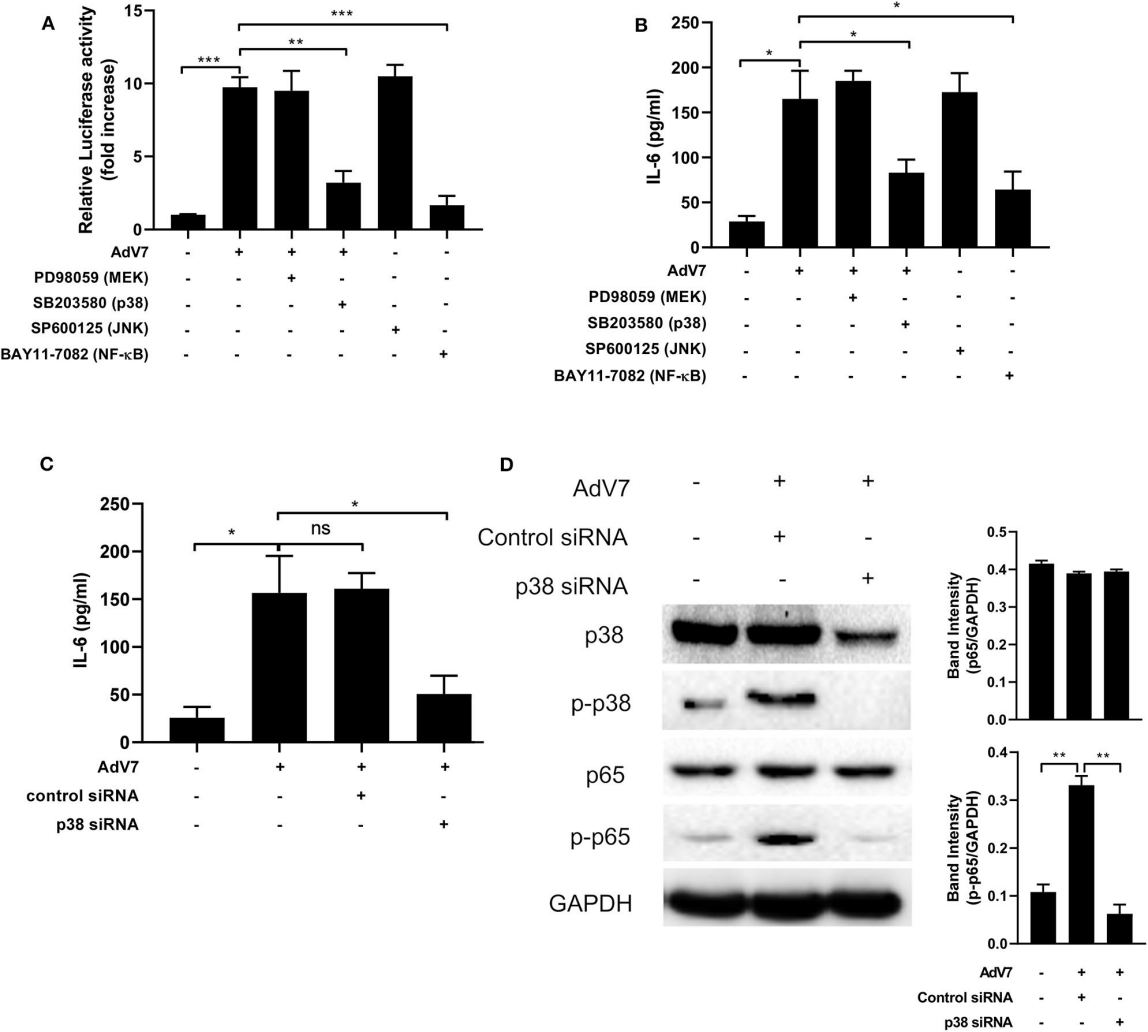
AdV 7 infection induces IL-6 expression *via* p38/NF-κB signaling pathway. **(A,B)** BEAS-2B cells were first **(A)** transfected with (-1,000/+11)IL-6-Luc and then **(A,B)** mock-infected or infected with 1 MOI AdV 7 and treated with different signaling pathway inhibitors. Twenty-four hours later, **(A)** cells were lysed and luciferase activity was measured, or **(B)** IL-6 concentration in the supernatant was measured by ELISA. Data shown are mean ± *SD* of three independent experiments with each condition performed in duplicate. *p < 0.05; **p < 0.01; ***p < 0.001. **(C,D)** BEAS-2B cells were first transfected with control siRNA or p38 siRNA, and then were mock-infected or infected with 1 MOI AdV 7. Twenty-four hours later, **(C)** IL-6 concentration in the supernatant was measured by ELISA, or **(D)** cells were lysed and the expression of p38, p-p38, p65, and p-p65 was determined by Western blot. **(C)** Data shown are mean ± *SD* of three independent experiments with each condition performed in duplicate. Ns, not statistically significant; *p < 0.05. **(D)** One representative result out of three is shown.

To examine which signaling pathway is involved in the AdV 7 infection induced IL-6 promoter transactivation, specific signaling pathway inhibitors targeting MEK (PD98059), p38 (SB203580), JNK (SP600125), and NF-κB (BAY11-7082) were used. As shown in Figure 5A and Supplementary Figure 3, NF-κB inhibitor expectedly reduced luciferase activity. In addition, cells with p38 inhibitor treatment also showed significant reduction in luciferase activity, indicating p38 pathway was involved in AdV 7 induced IL-6 promoter transactivation. To further confirm the involvement of p38 in the AdV 7 infection induced IL-6 upregulation, AdV 7 infection of BEAS-2B cells in the absence or presence of these specific signaling pathways inhibitors were performed and IL-6 expression was measured. Similar to the luciferase results, both p38 and NF-κB inhibitor significantly suppressed the expression of IL-6 (Figure 5B). The knock-down of p38 by siRNA also resulted in a similar reduction of IL-6 level in BEAS-2B cells with AdV 7 infection, further confirming that p38 is of importance in AdV 7-induced IL-6 upregulation (Figure 5C). Western blot analysis of BEAS-2B cells with or without AdV 7 infection in the presence or absence of p38 siRNA revealed that AdV 7 did not affect the expression of p38 but enhanced its phosphorylation (Figure 5D). In addition, downregulation of p38 by siRNA not only affected the p38 and p-p38 level, but also reduced p65 phosphorylation, implying the interconnection between p38 and NF-κB pathway in AdV 7-induced IL-6 upregulation (Figure 5D).

**Figure 6:**
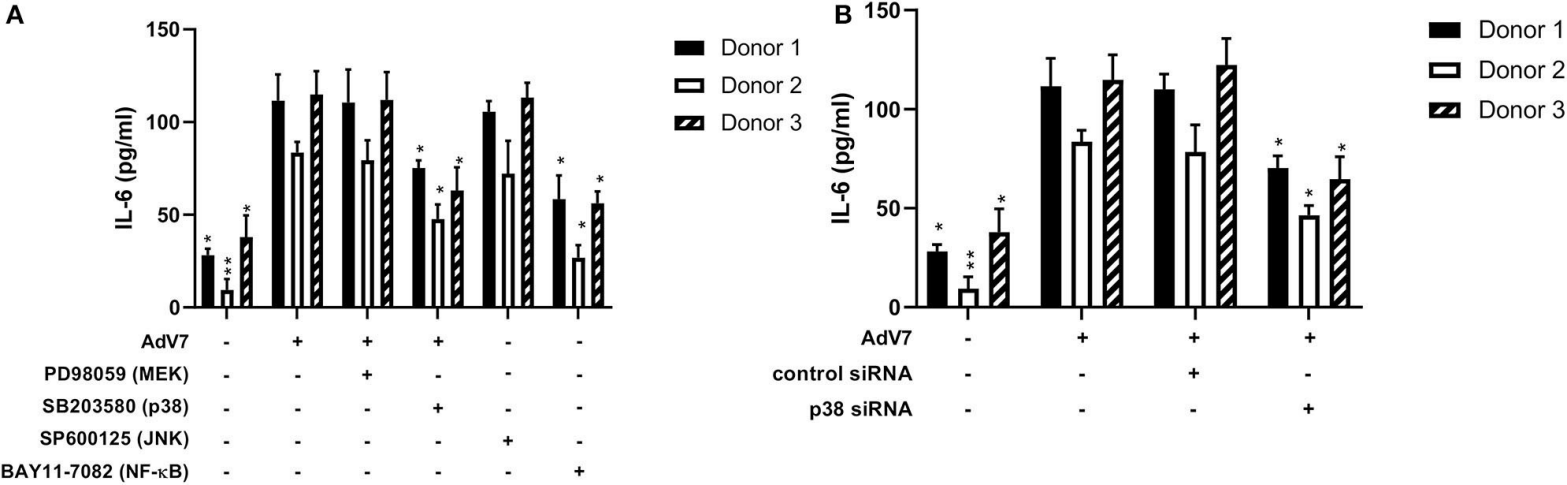
Blocking p38/NF-κB signaling pathway suppresses AdV 7-induced IL-6 expression. **(A,B)** primary hAEC cells were first **(A)** treated with signaling pathway inhibitors or **(B)** transfected with control siRNA or p38 siRNA, and then **(A,B)** infected with 1 MOI AdV 7. Twenty-four hours later, IL-6 concentration was quantified by ELISA. Data shown are mean ± *SD* of three independent experiments with each condition performed in duplicate. All statistical comparisons were made against cells with AdV 7 infection only. Ns, not statistically significant; *p < 0.05; **p < 0.01.

Our data on the BEAS-2B cell line showed that AdV 7 could increase IL-6 expression through p38/ NF-κB signaling pathway. To confirm this can be reproduced in primary airway epithelial cells as well, we investigated the impact of signaling pathway inhibitors and p38 siRNA on the expression of IL-6 in hAEC cells infected with AdV 7. As shown in Figure 6A, AdV 7 infection could enhance IL-6 expression, while p38 and NF-κB pathway inhibitor but not MEK and JNK inhibitor significantly suppressed IL-6 expression. Similarly, p38 siRNA also reduced IL-6 expression in AdV 7 infected hAEC cells (Figure 6B). To determine the inter-individual variances, primary airway epithelial cells from three different donors were used. As expected, cells from different donors responded to AdV 7 infection at different levels (Figures 6A,B). Despite the inter-donor difference, the trend observed in the primary cells was very similar to what was observed on BEAS-2B cells, although the IL-6 reduction was less profound in hAEC cells, confirming that AdV 7 infection induces IL-6 expression *via* p38/NF-κB pathway.

Taken together, our data in the current study have shown that AdV 7 infection triggers the production of a range of inflammatory cytokines in the airway in children, with IL-6 showing the highest elevation. And the induction of IL-6 expression by AdV 7 is mediated by IL-6 promoter transactivation through p38/NF-κB signaling pathway.

## Discussion

AdV is a very common virus infecting both adults and children, but usually AdV infection causes bad implications in children much more often than in adults. However, the research on this virus is underrepresented, with most of the AdV-related research being AdV-based vector studies. While the detailed mechanism underlying AdV-infection induced severe symptoms remains unclear, viral infection induced inflammation storm is believed to be one main cause. Several inflammatory cytokines have been proven to be elevated in AdV infected patients or cells, but unfortunately a more detailed cytokine profile upon AdV infection in patient airway and underlying mechanism remain to be further explored. In our current study, we have focused on AdV 7, a type usually causes severe pneumonia in children, and have analyzed a panel of inflammatory cytokines in the nasal wash of children infected with AdV 7. Our results have shown that most of the 13 tested cytokines are significantly increased, including IFN-α2, TNF-α, MCP-1, IL-6, IL-8, IL-10, IL-12p70, and IL-23. Among these elevated cytokines in AdV 7 infected patients, all but IL-10 are either pro-inflammatory cytokines or chemokines that responsible for chemoattracting inflammation-related immune cells to the site of infection, which together contribute to the inflammatory cytokine storm in the airway (23–28).

Unlike the other cytokines with elevation, IL-10 mainly functions as an anti-pro-inflammatory cytokine. While IL-10 has pleiotropic effects on immunoregulation and inflammation, it is capable of inhibiting the expression of a range of pro-inflammatory cytokines like IL-2, IL-3, and IFN-γ in Th1 cells and macrophages (29, 30). Under the environment of inflammation storm, the elevation of IL-10 seems to be surprising. Although the exact mechanism and biological relevance underlying IL-10 increase in AdV 7 infected patients remain to be investigated, it is possible that the immune system has detected the irregular inflammation in the airway and has tried but failed to restore the cytokine balance by upregulation of anti-inflammatory cytokines like IL-10, as elevation of IL-10 is very common in viral and bacterial infections (29).

Although many pro-inflammatory cytokines are increased in AdV 7-infected children, there are also a few showing no apparent increase (IFN-γ, IL-18, and IL-33) or even significant decrease (IL-1β and IL-17A) in our current study. Of these five cytokines, three of them (IL-1β, IL-18, and IL-33) belong to the same IL-1 superfamily. IL-1 family members are pro-inflammatory cytokines that are involved in the regulation of immune and inflammatory responses. IL-1β together with IL-23 can induce the expression of IL-17, IL-21, and IL-22 (31). IL-18 facilitates Th1 responses. Together with IL-12, IL-18 can induce IFN-γ production in T cells and NK cells (32). IL-33 is a cytokine that promotes Th2 response in Th, mast cells and eosinophils and basophils (33, 34). IL-17A belongs to the IL-17 family, which is mainly expressed by Th17 cells and plays an important role in mucosal immunity against infections (35). IFN-γ is a type II interferon and is critical for innate and adaptive immunity against viral and bacterial infections. Beside its immunostimulatory and immunomodulatory functions, IFN-γ can also directly inhibit viral replication (36). Given the importance of IL-17A and IFN-γ in the control of infections, the lack of production of these two cytokines in the airway of AdV 7 infected children may contribute to the onset of the inflammatory cytokine storm. However, it is important to remember that cytokines constitute a delicately regulated complex network and the increase or decrease of one cytokine might be regulated by several cytokines and may consequently affect the level and function of many other cytokines. Therefore, it is hard to accurately interpret the importance of the change of one or more cytokines.

The molecular mechanism underlying AdV infection induced cytokine production is also rarely studied. In the current study, we have chosen IL-6, the measured cytokine with the highest elevation in AdV 7 infected children and investigated the molecular mechanism in-depth on AdV 7-induced IL-6 expression. AdV infection triggers rapid and sharp elevation of inflammatory cytokines and chemokines. Such increase of inflammation is believed to be one of the main causes for AdV-induced clinical symptoms (13). Among many of the elevated cytokines/chemokines in AdV-infected individuals, IL-6 is reported to play an important role in the acute-phase innate response (37). Clinical trials using recombinant AdV have also suggested the interconnection between AdV-related cell cytotoxity and IL-6 (38, 39). In addition, in our study, we have discovered that nasal IL-6 level was higher in inpatients than in outpatients, suggesting a potential connection between nasal IL-6 and disease severity (Supplementary Figure 4). Given the importance and high elevation of IL-6, understanding the mechanism underlying AdV 7-induced IL-6 expression may be one of the key factors contributing to AdV 7-induced pathogenesis, and strategies down-regulating IL-6 expression may present a potential treatment to control AdV 7-induced severe clinical symptoms. Our results have shown that AdV 7 infection triggers the production of IL-6 by activating IL-6 promoter transactivation through p38/NF-κB signaling pathway. While this signaling pathway is very common in inflammatory response regulation and it is reported to be involved in the upregulation of many inflammation-related genes in various settings (40–42), it is not sure if this pathway also plays a role in the regulation of other cytokines in the airway under AdV 7 infection. Although beyond the scope of our current study, it would be very interesting to investigate the modulation mechanism of AdV-induced expression of inflammatory cytokines other than IL-6.

It has been previously described that, in many AdV-based viral vector studies, pro-inflammatory cytokines are induced in animals shortly after the systemic injection of AdV vectors and such response is independent of viral replication (43, 44). However, in our study, our data revealed that AdV 7-induced IL-6 expression was viral replication-dependent, as only live but not UV-inactivated virus could transactivate IL-6 promoter in airway epithelial cells (Supplementary Figure 2). Although further investigations are required, we believe that there are two possible explanations to the discrepancies between our data and data from previous AdV vector studies. First, most AdV-based studies have been done in animal models and immune cells including Kupffer cells and dendritic cells are the main cell types releasing pro-inflammatory cytokines like IL-6 (45). Whereas, in our study, we have used *in vitro* airway epithelial cell system. Therefore, viral structural proteins may not be able to trigger an IL-6 response in epithelial cells, which has been previously seen in animals. Second, different AdV types can cause distinctive type-specific responses and AdV vectors are usually not AdV 7-based (46). Consequently, it is possible that AdV 7 triggers a pro-inflammatory response that is quite different from the commonly used AdV-based vectors.

It is well-documented that different AdV types can cause distinctive type-specific clinical symptoms, however, it is not sure to what extent the inflammation cytokine profiles will be different from different types of AdV infection *in vivo*. A previous study has compared the expression of IL-6 and IL-8 in human bronchial epithelial cells infected with either AdV 3 or AdV 7. Although both AdV3 and 7 belong to the same subgroup (subgroup B), AdV 7 induces a more robust expression of both cytokines than AdV 3 (46). This implies a high possibility that the infection of different AdVs may cause quite different inflammatory cytokine production *in vivo*. Further studies addressing this question will be beneficial not only for the understanding of the disease pathogenesis, but also for the development of diagnostic and treatment strategies against different AdV infections.

There are a few limitations in our current study. First, AdV infection usually triggers the production of a wide range of inflammatory cytokines/chemokines. Although we have determined a 13-analyte panel containing the common inflammatory cytokines, this is still far from revealing the whole picture of AdV-induced inflammation. A more detailed inflammatory cytokine profile still remains to be further investigated. Second, we have not explored the correlation between nasal IL-6 with patient clinical symptoms. In-depth analysis of such correlation in a large cohort of patients will provide very valuable information for the diagnosis and treatment of AdV infection. Third, we have used traditional culture method for the culture of airway epithelial cells, instead of the air-liquid interface (ALI) culture system. It is reported that the ALI culture system can better resemble the *in vivo* airway epithelial cells. It would be interesting to compare the difference between the two cell culture systems in relation to AdV-induced inflammatory cytokine production profile.

## Data Availability Statement

The raw data supporting the conclusions of this article will be made available by the authors, without undue reservation.

## Ethics Statement

The studies involving human participants were reviewed and approved by Ethics Committee of Shenzhen Children's Hospital. Written informed consent to participate in this study was provided by the participants' legal guardian/next of kin.

## Author Contributions

LQ and YW designed the study and wrote the manuscript. LQ, YW, HW, and JD performed and analyzed the data. All authors read and approved the final manuscript.

## Conflict of Interest

The authors declare that the research was conducted in the absence of any commercial or financial relationships that could be construed as a potential conflict of interest.
