# Supplementary material for: Adenovirus 7 Induces Interlukin-6 Expression in Human Airway Epithelial Cells via p38/NF-κB Signaling Pathway
Source: Front Immunol. 2020 Sep 23;11:551413. doi: 10.3389/fimmu.2020.551413 (PMC7538593; doi:10.3389/fimmu.2020.551413)
Supplement: Supplementary file 1 [file Data_Sheet_1.DOCX]

Supplementary Material

# Supplementary Figures and Tables

## Supplementary Figures


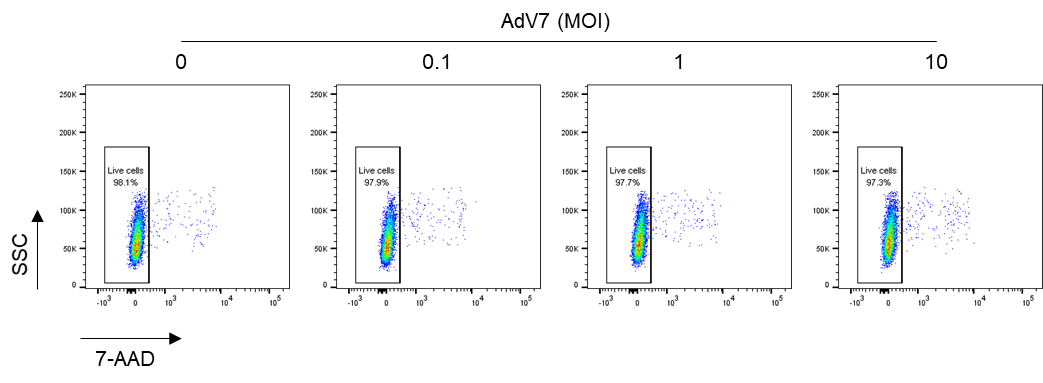


**Supplementary Figure 1.** The effect of AdV 7 infection on BEAS-2B cell viability. BEAS-2B cells were infected with ascendant doses of AdV 7 for 24 h and then cell viability was determined by 7-AAD staining.


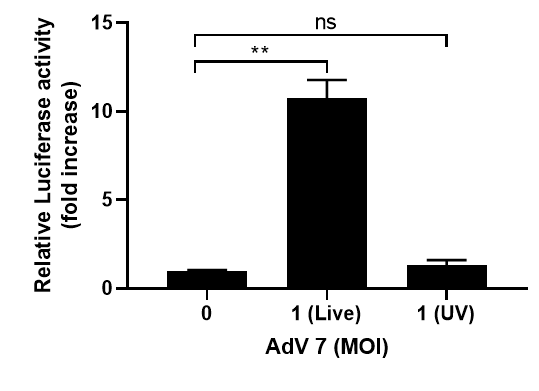


**Supplementary Figure 2.** Transactivation of IL-6 promoter by live and UV-inactivated AdV7. BEAS-2B cells were first transfected with (-1000/+11)IL-6-Luc, and then either mock-treated, or treated with 1 MOI of live or UV-inactivated AdV 7. Twenty-four hours later, cells were lysed and luciferase activity was measured. Data shown are mean ± SD of three independent experiments with each condition performed in duplicate. *, p < 0.05; **, p < 0.01.


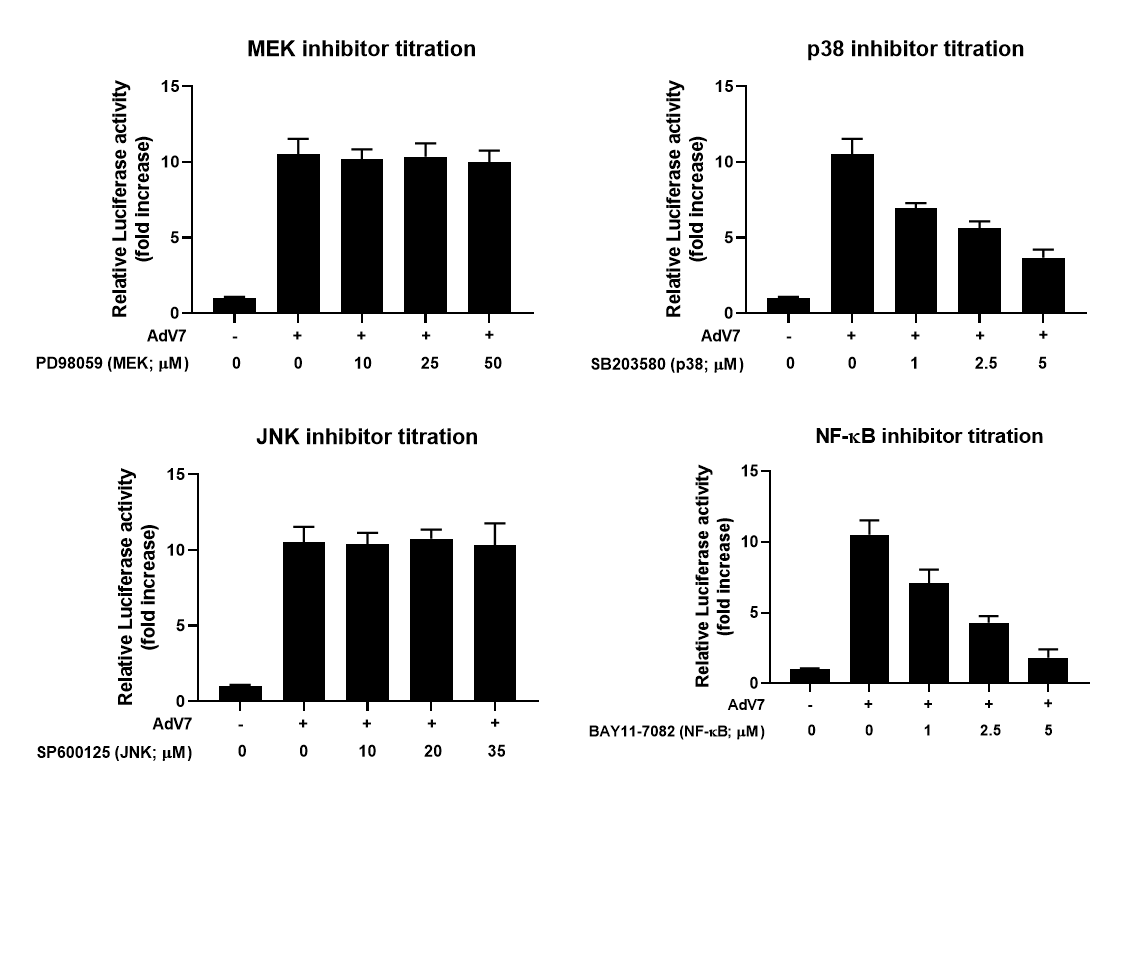


**Supplementary Figure 3.** Dose titration of signaling pathway inhibitors. BEAS-2B cells were first transfected with (-1000/+11)IL-6-Luc and then mock-infected or infected with AdV 7 and treated with different signalling pathway inhibitors. Twenty-four hours later, cells were lysed and luciferase activity was measured. Data shown are mean ± SD of three independent experiments with each condition performed in duplicate.


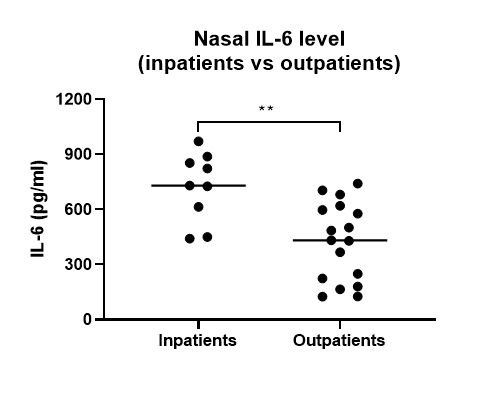


**Supplementary Figure 4.** Higher nasal IL-6 level in inpatients than in outpatients. Nasal samples from AdV 7 infected children were harvested and IL-6 was measured by BioLegend’s LEGENDplex bead-based immunoassay. **, p < 0.01.
